# Supplementary material for: Complexome profiling of the Chlamydomonas psb28 mutant reveals TEF5 as an early PSII assembly factor
Source: Plant Cell. 2025 Mar 17;37(6):koaf055. doi: 10.1093/plcell/koaf055 (PMC12164590; doi:10.1093/plcell/koaf055)
Supplement: koaf055_Supplementary_Data [file koaf055_supplementary_data.zip › psb28_interactive.zip!/plots/intensity/qualityPlots/peptides/_Cre13.g564050.t1.1.html]

### Cre13.g564050.t1.1

Trivial name:   
SeqCoverage [median]: 17.90  
Mass: 28.61 kDa  
  
 

| Sequence | MissedCleavage | Mass [Da] | wtIntensity | muIntensity | Score |
| --- | --- | --- | --- | --- | --- |
| AATESFR | 0 | 780.38 | 209.84 | 0.00 | 76.85 |
| GPAPEPSPVR | 0 | 1005.52 | 0.00 | 715.13 | 60.59 |
| VRYDSPDGNTVLSVLVR | 1 | 1889.00 | 505.26 | 0.00 | 31.80 |
| VVAGSVLQVALPPR | 0 | 1404.85 | 0.00 | 703.26 | 54.52 |
|  |  |  | 715.1 | 1418.4 |  |
